# Dual Role of MiR-21-Mediated Signaling in HUVECs and Rat Surgical Flap under Normoxia and Hypoxia Condition

**DOI:** 10.3390/ijms18091917

**Published:** 2017-09-07

**Authors:** Chih-Hau Chang, Meng-Chi Yen, Ssu-Hui Liao, Yu-Ling Hsu, Chung-Sheng Lai, Yur-Ren Kuo, Ya-Ling Hsu

**Affiliations:** 1 Graduate Institute of Medicine, College of Medicine, Kaohsiung Medical University, Kaohsiung 807, Taiwan; igor8301023@gmail.com (C.-H.C.); s0970215575@gmail.com (S.-H.L.); l32861wa@yahoo.com.tw (Y.-L.H.); 2 Division of Plastic and Reconstructive Surgery, Kaohsiung Medical University Hospital, Kaohsiung 807, Taiwan; chshla@kmu.edu.tw (C.-S.L.); kuoyurren@gmail.com (Y.-R.K.); 3 Department of Emergency Medicine, Kaohsiung Medical University Hospital, Kaohsiung Medical University, Kaohsiung 807, Taiwan; yohoco@gmail.com; 4 Faculty of Medicine, College of Medicine, Kaohsiung Medical University, Kaohsiung 807, Taiwan

**Keywords:** hypoxia, angiogenesis, MicroRNA-21 (miR-21), SMAD7

## Abstract

Restoring sufficient vascularity of the ischemia/hypoxia flap is always the critical issue in flap surgeries. In a previous studies microRNA-21 (miR-21) expression was upregulated after rat skin flap surgery. MiR-21 has been reported to be induced by hypoxia and the function of miR-21 involves in the process of angiogenesis. However, the precise regulatory mechanisms in miR-21-mediated pathways are still unclear. These issues were investigated via in vitro and in vivo experiments in this study. In human umbilical vein endothelial cells (HUVEC), the expression of hsa-miR-21-5p was induced after hypoxic culture and the induction of hsa-miR-21-5p was suppressed after sequential normoxic culture. Moreover, transfection of hsa-miR-21-5p mimic enhanced tube formation capacity in normoxia, but attenuated it in hypoxia. Furthermore, bioinformatic analysis suggested that SMAD7 was a predicted target of hsa-miR-21-5p. Our results demonstrated the effect of hsa-miR-21-5p was different on SMAD7 expression in normoxia and hypoxia. In rat skin flaps, blockage of miR-21-5p significantly increased angiogenesis via analysis of color laser Doppler imaging and repressed SMAD7 expression in ischemic skin tissue. Our study showed the opposite effect of miR-21-5p mediating angiogenesis in normoxia and hypoxia, providing important implications regarding the design of novel miRNA-based therapeutic strategies in flap surgeries.

## 1. Introduction

Skin flap transfer is a basic plastic surgery method used for wound repair and reconstruction [1]. However, the need for extended design of flap to reconstruct huge defects is quite usual, and this implies a high risk to jeopardize whole flap survival, resulting in failure due to possible persistent ischemia, hypoxia, or ischemia-reperfusion injury, which is a complex phenomenon accompanied with a lack of blood supply results in hypoxia/anoxia and then following by resumption of blood flow [2,3]. In the distal part of the flap, poor blood flow within the flap itself is observed in some clinical cases. Loss of microvascular function leads to insufficient oxygen supplement and tissue damage, such as partial flap necrosis of the free or pedicle flaps [3]. Overcoming such a scenario of partial failure caused by ischemia injury is always a challenge to all microsurgeons.

Micro-RNAs (miRNAs) belong to noncoding single-stranded RNAs of 21–25 nucleotides that can regulate posttranscriptional gene expression [4]. They have been identified as being involved in the ischemia-reperfusion injury of the brain [5], heart [6], kidney [7], and vessels [8]. Previous studies showed that expression of four miRNAs, including miR-96, miR-193-3p, miR-210, and miR-21, were correlated with the skin flap model in rat [9]. However, the detailed molecular mechanism underlying the effect of miRNAs remains undetermined.

The therapeutic strategy of preventing from the flap loss has been focused on improving blood supply in the compromised tissue [10,11]. Several studies have been demonstrated that miR-21 is involved with the regulation of ischemia or ischemia-reperfusion injury in various tissues and associated processes such as angiogenesis and cell survival [12,13,14]. In the present study, we investigated the expression of miRNAs in vascularity-insufficiency flap of rat, regulatory the role of the miRNA and miRNA-mediated signaling pathway in human umbilical vein endothelial cells (HUVECs) and in a rat surgical flap model.

## 2. Results

### 2.1. Indocyanine Green (ICG) and Laser Doppler Quantificate the Insufficient Vascularity of the Distal Skin Flap

**Figure 1:**
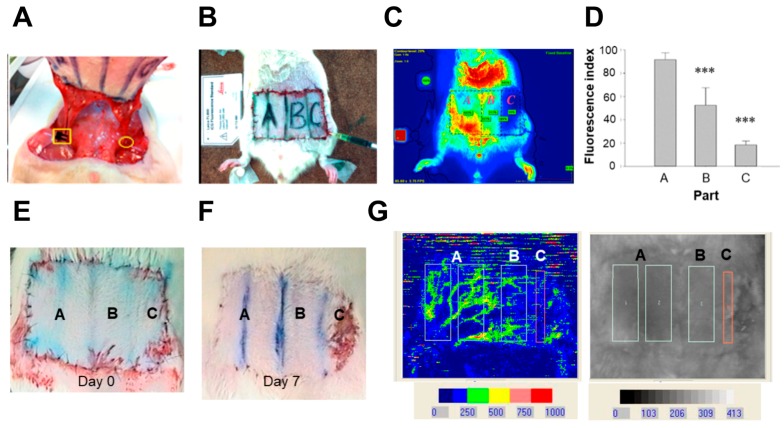
Evaluation of blood flow via indocyanine green (ICG) fluorescence and laser Doppler imaging after flap surgery. (**A**) The right epigastric vessel was ligated by Nylon 5-0 (yellow circle). The yellow square indicates the use of bulldog badge clamp over the left epigastric vessel for 1 h for ischemic manipulation. The manipulation mimics free flap surgery and partial ischemic failure. The rat subjected to ICG fluorescence imaging under (**B**) white light and (**C**) ICG fluorescence imaging. All flaps were divided into Parts A, B, and C. Part A indicates sufficient-vascularity, Part B indicates borderline-vascularity, and Part C indicates insufficient-vascularity; (**D**) Quantification of ICR imaging (*n* = 4). The images of rat skin at (**E**) day 0, and (**F**) day 7 post-operation. The Part B was shrunk and Part C showed the partial necrosis of the skin flap at day 7 (the red rectangle); (**G**) The region of LASER Doppler quantification. Data is shown as mean ± standard error of the mean (SEM). *** *p* < 0.001 significantly different from the Part A.

To address the blood flow after flap surgery, a rat animal model was used. Figure 1A showed the flap surgery. One side of epigastric vessel was ligated and the blood flow was completely blocked. The blood flow was stop by a bulldog badge clamp for 1 h in the other side and then the clamp was removed. The manipulation respectively mimics ischemia and ischemia-reperfusion condition. The abdomen skin flap was shaved and marked by a fixed template (area size: 6 × 3.6 cm). It was divided equally into four parts as the vascularity decreasing fashion. Part A contained half of the flap and the base on which the vessel was manipulated. Part B contained the quarter area of the flap next to Part A. Part C contained the last quarter of the flap base on which the vessel was totally ligated (ischemia condition) (Figure 1B, day 0 after operation). The ICG imaging revealed that Part A of the skin flap showed sufficient-vascularity, Part B of the skin flap showed borderline-vascularity, and Part C of the skin flap showed insufficient-vascularity among the rat skin flap (Figure 1C). The quantitative results showed the blood flow in Parts A was significantly higher than that in Part B and Part C (Figure 1D). Compared to the skin at day 0, Part B showed the area was shrunk and Part C showed the partial necrosis at day 7 post-operation (Figure 1E,F). In Figure 1G, the results of laser Doppler velocimetry showed that relatively low blood flow in Part C.

### 2.2. Evaluation of Hsa-MiR-21-5p Expression under Hypoxia Stress in HUVEC

**Figure 2:**
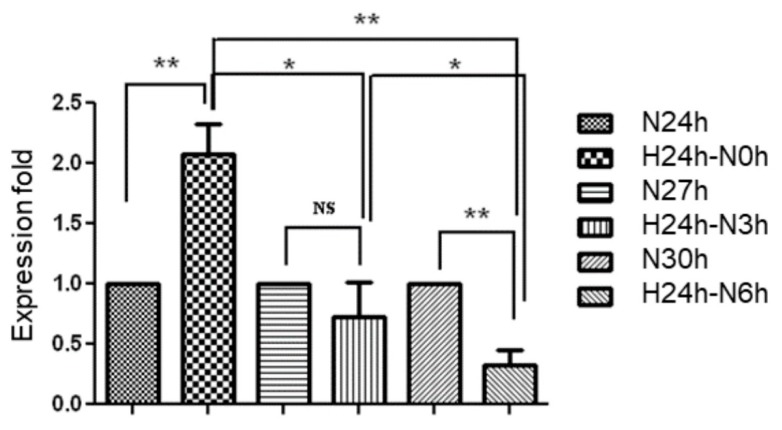
Evaluation of hsa-miR-21-5p expression in human umbilical vein endothelial cells (HUVEC). HUVEC was cultured at normoxia (N) and hypoxia (H) condition for different incubation periods including normoxia for 24 to 30 h (N24h to N30h), and hypoxia for 24 h then normorxia for 0 to 6 h (H24h–N0h to N6h). Data is shown as mean ± SEM from three independent experiments. * *p* < 0.05, ** *p* < 0.01, significant difference between two groups; NS, no significant difference.

Positive correlation between ischemia-reperfusion injury in the skin flap of rat and four miRNAs (miR-21, miR-96, miR-193, and miR-210) has been demonstrated in previous studies [9]. In addition, miR-21 involves in hypoxia-induced pulmonary vascular remodeling and angiogenesis in renal tissue [15,16]. To further investigate the function of miR-21-5p in endothelial cells, the human umbilical vein endothelial cell, HUVEC, was cultured in normoxia (20% oxygen) or hypoxia (1% oxygen) condition for 24 h. Since the expression level of miR-21 was slightly suppressed at 2 h reperfusion after ischemia in the rat flap [9], we hypothesized that the miR-21 level was changed in a short period of time under hypoxia and sequential normoxic exposure. The hypoxia-cultured HUVEC was then sequentially exposed to normoxia for 3 or 6 h in order to mimic the transition of hypoxia and normoxia. In Figure 2, the expression of miR-21 in hypoxia-cultured HUVEC was significantly higher than that in normoxia-cultured HUVEC. Furthermore, re-exposure to normoxia significantly decreased hypoxia-induced miR-21 expression in a time-dependent manner. These results indicated that the expression of miR-21 was induced by hypoxia in HUVEC.

### 2.3. Investigating the Role of Hsa-MiR-21-5p in Tube Formation Capacity in HUVEC

**Figure 3:**
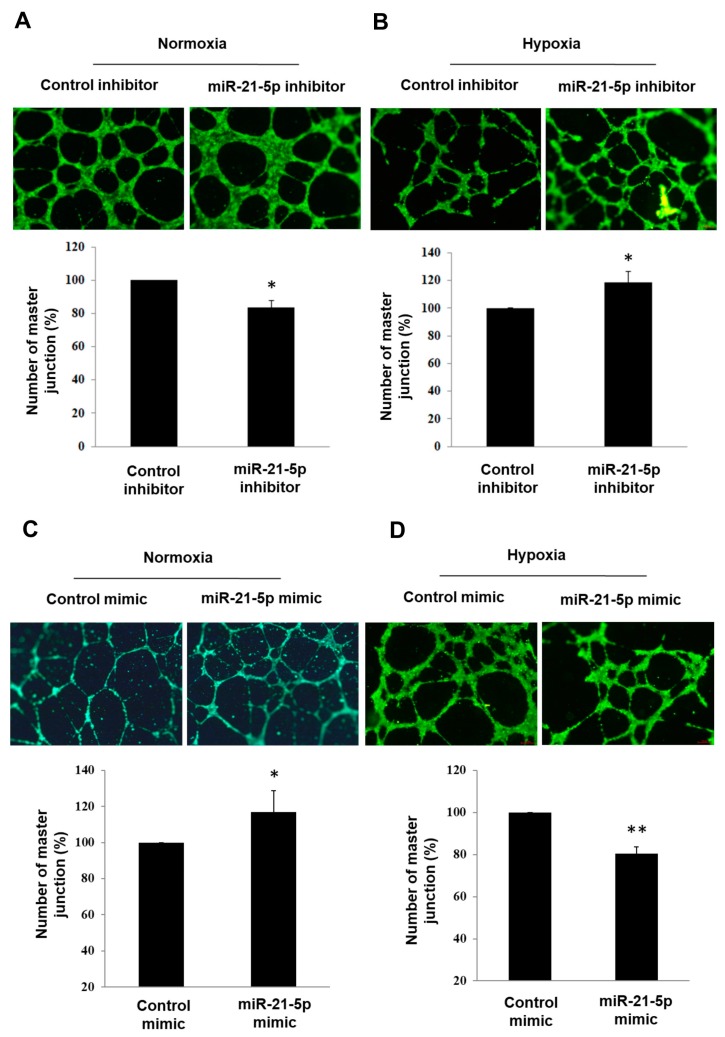
The effect of hsa-miR-21-5p on tube formation assay in HUVEC. HUVEC was transfected with 100 nM of mimic-miR-21-5p or mimic control and then cultured at (**A**) normoxia and (**B**) hypoxia for 24 h. Images of tube formation assay were shown in upper panel and quantitative results were shown in lower panel. The relative number of master junction was shown in Y-axis of quantification results (control group was set to 100%). HUVEC was transfected with 100 nm of inhibitor-miR-21-5p or inhibitor-control at (**C**) normoxia and (**D**) hypoxia for 24 h. The bar graph was shown as mean ± SEM from three independent experiments, * *p* < 0.05, ** *p* < 0.01, significant difference between two groups. Scale bar = 68 μm.

In several types of cancer, miR-21 involves in regulation of angiogenesis [17,18,19]. Moreover, miR-21 was reported to play a pro-angiogenic role in retinal microvascular endothelial cells in previous studies [20]. Overexpression of miR-21 enhances vascular endothelial growth factor-induced tube formation capacity in HUVEC [21]. This might suggest miR-21 is pro-angiogenic in endothelial cells. In Figure 3A, transfection of hsa-miR-21-5p inhibitor significantly decreased tube formation capacity in normoxia condition. Interestingly, opposite effect of hsa-miR-21-5p inhibitor was observed in hypoxia (Figure 3B). When HUVEC was transfected with hsa-miR-21-5p mimic, the tube formation capacity was enhanced in normoxia condition and was suppressed in hypoxia (Figure 3C,D). It suggested that the function of hsa-miR-21-5p on tube formation capacity was opposite in normoxia and hypoxia condition.

### 2.4. Investigation of Hsa-MiR-21-5p-Mediated Signaling Molecules under Hypoxia Stress in HUVEC

**Figure 4:**
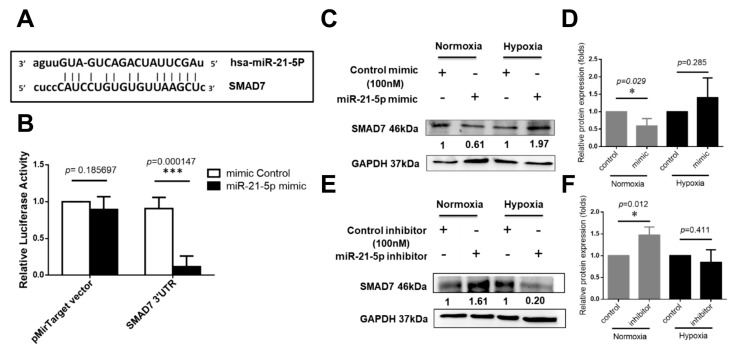
SMAD7 is a direct target of hsa-miR-21-5p. (**A**) The 3′-UTR sequences of SAMD7 is cloned at the 3′ end of luciferase reporter gene in the pMirTarget plasmid. The predicted binding site of hsa-miR-21-5p on the region of 3′-UTR sequence of SMAD7 (SMAD7 3′-UTR) was shown. The “seed sequences” were indicated by lowercase; (**B**) Luciferase reporter assay. HEK293 cells were transfected with mimic-miR-21-5p or mimic control while transfection of pMirTarget plasmid or SMAD7 3′-UTR plasmid. *** *p*  <  0.001, significant difference between two groups; (**C**) The effect of mimic-miR-21-5p on SMAD7 protein expression and (**D**) quantification of relative SMAD7 expression; (**E**) the effect of inhibitor miR-21-5p on SMAD7 protein expression and (**F**) quantification of relative SMAD7 expression. * *p* < 0.05, *** *p* < 0.001, significant difference between two groups.

To investigate the putative miR-21 targets, four online microRNA target databases including TargetScan (http://www.targetscan.org/vert_71/) [22], PicTar (http://pictar.mdc-berlin.de/) [23], microRNA.org (http://www.microrna.org) [24], and miRDB (http://mirdb.org/) [25], the predicted results suggested that SMAD7 are potential targets of miR-21. Similar results are in accordance with previous studies [26,27,28,29]. The predicted miR-21 targeting site on SMAD7 was shown in Figure 4A. The reporter assay demonstrated that overexpression of miR-21 could bind to the 3′-UTR of SAMD7 (Figure 4B). In HUVEC cultured at normoxia condition, miR-21 expression suppressed protein expression of SMAD7 (Figure 4C,D). In contrast, expression of miR-21 was reversely correlated with SMAD7 protein expression in hypoxia (Figure 4E,F). It might imply that the miR-21/SMAD7 involves in different regulatory mechanism when cells were cultured at normoxia and hypoxia.

### 2.5. Investigating the Effect MiR-21-5p on Blood Flow in the Skin Flap

**Figure 5:**
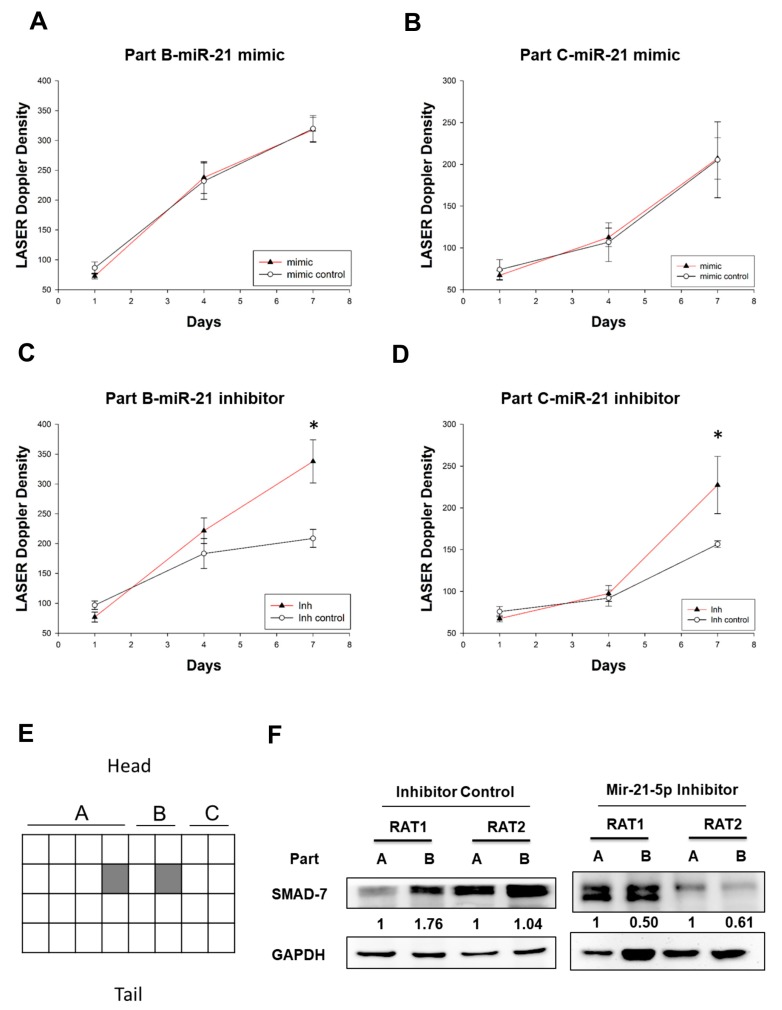
Treatment of inhibitor miR-21-5p enhances blood flow in the rat model. The density of laser Doppler angiography image was illustrated at day 7 post operation (shown as Figure 1F). Meanwhile, each square was outlined and calculated as the mean of the angiogenesis density of the corresponding region (including Part B and Part C). Angiogenesis of (**A**) Part B and (**B**) Part C of skin flap after subdermal local injection of mimic miR-21. Angiogenesis of (**C**) Part B and (**D**) Part C of skin flap after subdermal local injection of inhibitor miR-21 into Part B. The results were analyzed by laser Doppler on day 1, day 4, and day 7 post-miR injection, * *p*  <  0.05, significant difference between two groups at day 7 post miR injection, * *p*  <  0.05, significant difference between two groups at the same day (*n* = 6 in each group); (**E**) The illusion of rat skin. The Part A and Part B was divided into 16 and 8 regions, respectively. The protein of rat skin was collected from the shading region of Part A and Part B in miR-21-5p inhibitor- and miR-21-5p inhibitor control-treated rat at day 7 after flap operation; (**F**) SMAD7 protein expression in rat skin. RAT1 and RAT2 indicate two independent rat from each group (*n* = 2). Symbol mimic: miR-21-5p mimic; Inh: MiR-21-5p inhibitor; Inh control: Inhibitor control.

Since miR-21 showed anti-angiogenesis capacity in vitro in hypoxia (Figure 3D), we investigated whether delivery of miR-21-5p enhanced blood flow in an animal model. Ischemia preconditioning procedure with the miR-21-5p mimic and mimic control, or miR-21-5p inhibitor and inhibitor control, were administered half an hour before surgery with a dose of 33 ng/µL and total 1 mL divided into four parts, respectively, were injected subdermal within Part B (the image was shown in Figure 1E,F). Seven days after miRNAs injection, there was no effect of miR-21-5p mimic in both Part B and Part C in skin flap (Figure 5A,B). However, miR-21-5p inhibitor significantly increased the skin blood flow of Part B and Part C at day 7 post-operation (Figure 5C,D). These results suggested that down-regulation of miR-21-5p could enhance the angiogenesis in skin flaps. To investigate the effect of miR-21-5p inhibitor in vivo, the Part A and Part B of rat skin was collected at day 7 post flap surgery (Figure 5E). Part C was not collected because of necrosis and shrink at day 7. In the miR-21-5p inhibitor treated rat skin, the expression of SMAD7 was suppressed in Part B which was a relatively ischemic region when compared to that in Part A (Figure 5F). This evidence might suggest that inhibition of miR-21-5p in hypoxia condition enhances angiogenesis via down-regulation of SMAD7.

## 3. Discussion

Ischemia-induced injury is a problem in microvascular surgery, especially for free tissue transfer and replantation of mangled amputated body parts. Meanwhile, restoration of blood flow at reperfusion following ischemia is essential for any flap survival. MicroRNAs involve in the endothelial proliferation, migration, and tube formation [30,31,32]. In addition, various hypoxia-induced microRNAs are associated with angiogenesis [33]. Since the expression of miR-21 is correlated with surgical flap [9], we examined whether miR-21 involved in angiogenesis in rat skin flap which is a hypoxic environment. Our results showed that the miR-21 play different roles in angiogenesis under normoxia and hypoxia and SMAD7 might be an important regulator. Furthermore, inhibition of miR-21 increased blood flow in skin flap. This suggests that miR-21 suppresses angiogenesis in human endothelial cell and rat skin flap in the hypoxia.

Ischemia/hypoxia-induced angiogenesis is an important process in the repair of the ischemic injury through restoration of a normoxic environment in the impacted tissue. Several studies have shown that angiogenesis can be induced in various organs following ischemia such as myocardium, kidney, and brain [34,35]. The endothelial cell has been induced the proliferation, migration, and new vasculature formation occurred 2–14 days after renal ischemic injury [36]. Lack of miRNAs, such as Dicer and Drosha, have been reported to abolish the generation and migration of capillary endothelial cells and alter factors that regulate angiogenesis, suggesting that miRNAs has a dramatic impact on angiogenesis [37]. The pro-angiogenic role of miR-21 was demonstrated in some reports [20,21]. Furthermore, miR-21 not only enhances tube formation capacity, but also cell proliferation and migration in endothelial cells [20,30]. In the present study, miR-21 results in deceasing tube formation capacity in HUVEC in hypoxia and leads to anti-angiogenesis in the rat flap. Although the effect of miR-21-5p on cell migration and proliferation was not evaluated in the present study, our data might imply that miR-21-5p inhibitory effect on angiogenesis in hypoxia condition.

To explore the putative mechanism for the involvement of miR-21 in the hypoxia induction of HUVECs tube formation, we focus on the identification of target genes potentially regulated by miR-21. Our present results show that miR-21 directly targets the 3′-UTR of SMAD7. SMAD7 is reported as a target of miR-21 in other studies [26,27,28,29]. Additionally, our data revealed that the protein expression of SMAD7 is regulated by miR-21 in normoxia and hypoxia. Gene transfer of SMAD7 decreases peritoneal angiogenesis [38]. An emerging study indicates that treatment of metformin (an anti-diabetic drug) reduces miR-21 expression, increase SMAD7 expression, and then abolished cells proliferation, migration, tube formation in HUVEC [27]. These evidences suggest that miR-21 induction results in decreasing SMAD7 expression and enhancing angiogenesis. Our results are similar with these studies in normoxia, but the opposite effect of miR-21 is observed in hypoxia. Hypoxia-inducible factors-1α (HIF1α) is a critical molecule that respond to hypoxia. In other cell types, miR-21 expression contributes to the up-regulation of HIF1α and enhances angiogenesis in human umbilical cord blood-derived mesenchymal stem cells and kidney cells [39,40]. In the present study, the function of miR-21 is related to anti-angiogenesis in hypoxia. It might imply that the regulatory mechanism between HIF1α and miR-21-5p in endothelial cells is different from other cell types. The role of HIF1α should be investigated in the future.

SMAD7 expression is suppressed when delivery of miR-21-5P inhibitor in hypoxia in vitro and in vivo. It might imply that SMAD7 is regulated by some unknown signaling pathways in hypoxia condition. According to the previous studies, the other direct targets of miR-21, such as PTEN, AKT, and ERK, have been reported, these molecules involve in pro-angiogenesis phenotypes [27,41]. In addition, transforming growth factor-β is also an important factor to regulate miR-21/SMAD7 signaling pathways in other types of cells [26,42]. In other pathophysiological condition related to ischemia injury/reperfusion injury, miR-21 is related to protective effect (such as increasing angiogenesis and reducing apoptosis), and damaging effect (such as induction of fibrosis and inflammation) [2]. The angiogenesis-inhibitory effect of miR-21 is also unclear in other cell types. The detailed regulatory mechanism in endothelial cells needs to be further investigated in the future.

## 4. Materials and Methods

### 4.1. Cell Culture

Human umbilical vein endothelial cells (HUVECs, ATCC^®^ CRL-1730™) and HEK293 (ATCC^®^ CRL-1573™) were obtained from the American Type Culture Collection (Manassas, VA, USA). HUVEC was cultured in EGM-2 MV BulletKit Medium (Lonza, supplied with EGM-2 SingleQuot Kit Suppl. and Growth Factors (Lonza) which contains 2% FCS (fetal calf serum), hydrocortisone, bFGF (basic fibroblast growth factor), R3-IGF-1 (long R-insulin-like growth factor), ascorbic acid, hEGF (human endothelial growth factor), GA-1000 (gentamicin sulfate and amphotericin-B), at 37 °C. HEK293 was cultured in Eagle’s minimum essential medium (Lonza) supplied with 10% fetal bovine serum at 37 °C.

### 4.2. Quantification of MiR-21-5p

RNA was extract via TRIzol Reagent (Invitrogen, Carlsbad, CA, USA) and complementary DNA was reverse transcribed via Mir-X miRNA First-Strand Synthesis kit (Clontech Laboratories, Mountain View, CA, USA) according to the manufacturer’s instruction. The level of miRNA was analyzed using Fast SYBR-Green Master Mix (Applied Biosystems, Foster City, CA, USA) on StepOne Plus Real-Time PCT System (Applied Biosystems). The miR-21-5p was detected via specific primer: 5′-TAGCTTATCAGACTGATGTTGA-3′ and mRQ 3′ primer (provided by Mir-X miRNA First-Strand Synthesis kit). Normalization was performed with a small nuclear RNA U6 (provided by Mir-X miRNA First-Strand Synthesis Kit). The ΔΔ*C*_t_ method was used for calculating the relative miR-21-5p expression and the relative expression of each control group was always set to 1.

### 4.3. Tube Formation Assay

Before tube formation analysis, HUVEC cells were transfected with 100 nM of miR-21-5P mimic, miR-21-5P inhibitor, or control miR (Dharmacon, Lafayette, CO, USA) by DharmaFECT Transfection Reagent 4 (Dharmacon) in complete growth medium, and then were incubated for 24 h in normoxia (5% CO_2_, 20% O_2_, at 37 °C) and hypoxia (5% CO_2_, 95% N_2_, 1% O_2_, at 37 °C). After 24 h, about 4 × 10^5^/well HUVEC cells were seeded in 48 well plate which was pre-coated with growth-factor-reduced Matrigel (BD Biosciences, Mississauga, ON, Canada). The result was measured at 8 h after incubation in normoxia and hypoxia condition. Each well was washed, fixed, stained with 1 µg/mL of Calcein AM (Molecular Probes, Eugene, OR, USA) for 30 min at 37 °C, and viewed through a microscope. Total tube length was measured in three fields (10×) using Image J software version 1.51 (National Institutes of Health, Bethesda, MD, USA).

### 4.4. Western Blotting

After appropriate treatment in each experiment, cells were lysed in radioimmunoprecipitation assay buffer (RIPA) (Millipore, Billerica, MA, USA) on ice for 30 min. The total cell lysate was then collected after centrifugation at 4 °C, 12,000× *g* for 15 min. Equivalent amount of protein was loaded and separated by sodium dodecyl sulfate-polyacrylamide gel electrophoresis (SDS-PAGE) (10%) and transferred to polyvinylidene difluoride membranes. The membrane was blocked in 5% non-fat dry milk for 1 h. After blocking, membranes were incubated with primary antibodies with against SMAD7 (1:1000, Cat.No. ST1625, Millipore) and GAPDH (1:5000, Cat.No. MAB374, Millipore), and the secondary antibody was horseradish peroxidase-conjugated goat anti-mouse IgG antibody raised against mouse IgG. The results were detected using an enhanced chemiluminescence substrate (Millipore) on an imaging capture system (Alpha Innovation, Kent, UK). Quantification of protein expression was performed via Image J software version 1.51 (National Institutes of Health).

### 4.5. Reporter Assay

The complete sequence of SMAD7 3′ untranslated region (UTR) was constructed into the pMirTarget vector (Origene, Rockville, MD, USA). The plasmid containing the putative binding sequence of miR-21-5p “ATAAGCTA” is named as “SMAD7 3′UTR”. For luciferase assay, 50 nm of control miRNA or miR-21-5p mimics, 0.8 µg of pMirTarget vector containing SMAD7 3′ UTR, and 0.08 µg of pRL renilla luciferase control reporter vector (Promega, Madison, WI, USA) were co-transfected into HEK-293 cells in 96-well plates for 48 h. Cell were harvested and analyzed using the dual-luciferase reporter assay (Promega) according to the manufacturer’s instruction.

### 4.6. Rat Skin Flap Surgery

All animal procedures were performed using male Sprague-Dawley (SD) rats obtained from the National Science Council and approved by the Institutional Animal Care and Use Committee of Kaohsiung Medical University (approval code: 104228, approval date: 23 May 2016). The experiments were performed on adult male SD rats weighing 250–300 g. Rats were anesthetized with 50 mg/kg of sodium pentobarbital by intraperitoneal injection prior to surgery in the prone position and maintained with 5 mg of sodium pentobarbital in each subsequent shot, if needed. The bilateral epigastric pedicle nutritious flap was modified from our previous study [9]. Then, the first side of femoral pedicle was totally ligated with Nylon 5-0 as the extended-designed flap and the other side of femoral pedicle was clamped temporally for 1 h for ischemic manipulation to mimic free flap surgery. All flaps were divided into Parts A, B, and C (Part A: Sufficient-vascularity zone near the clamped part; Part B: Borderline-vascularity zone closer to the clamped part; Part C: Insufficient-vascularity zone far from the clamped part).

### 4.7. Assessment of Blood Flow in Rat Flap

The blood flow in rat skin flap was assessed by indocyanine green (ICG)-based imaging system (SPY syetem; Novadaq Technologies; Toronto, ON, Canada) and moorLDI2 laser Doppler imager (Moor Instruments, Axminster, UK). For ICG analysis, the ICG pattern was determined after one-minute ICG flow perfusion with ICG dosage 1.5 mg/kg systemically. For laser Doppler analysis, a moving mirror directs a beam of coherent red light generated by a 633-nm helium-neon laser on the skin. Computer-controlled rotations of the mirror around two perpendicular axes allow the scanning of a square region. The surface of the scanned area (lower abdomen of the rat) can be covered with fixed angular amplitudes and skin-mirror distance. From the analysis of the backscattered Doppler-shifted light, microvascular blood flow in each of up to 256 × 256 adjacent spots (“pixels”) is calculated, with a computation time of 4 ms/pixel. Thus, at full spatial resolution, a complete scan is obtained in 5 min. The final result is a computer-generated, color-coded image of the spatial distribution of microvascular blood flow. The blood flow could be calculated later by summing the pixel values in an arbitrarily shaped region of interest within the scanned area, using the Moor LDI software (Moor Instruments).

### 4.8. Delivery of MiRNA

The miR-21 mimic and miR-21 inhibitor were injected half an hour before surgery via in vivo-jetPEI reagent (Cat. No. 201-50G, Polyplus, Strasbourg, France) according to the manufacturer’s instruction. The miRNA was injected with a dose of 33 ng/µL and total 1 mL (33 µg miRNAs) divided into four parts, respectively, for subdermal injection within the border group of the flap.

### 4.9. Statistical Analysis

Differences between two independent groups were analyzed by the Student’s *t*-test. Comparisons between three groups were performed using ANOVA with Dunnett’s test. The significant difference (*p* < 0.05) between each group was considered. All calculations were carried out using the software SigmaPlot 12.5 (Systat Software, Inc., San Jose, CA, USA).

## 5. Conclusions

**Figure 6:**
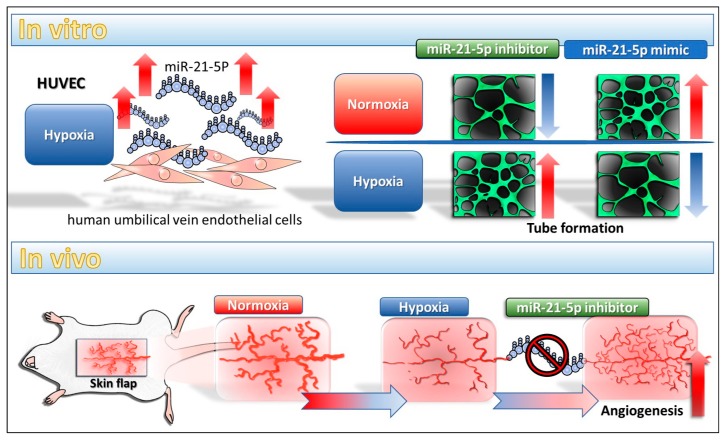
The proposed model of miR-21-5p in vitro and in vivo.

Our study showed the miR-21-5p play an anti-angiogenesis role in hypoxia condition in vitro and in vivo. Inhibition of miR-21-5p enhances angiogenesis and is correlated with down-regulation of SMAD7 in ischemic rat skin. In addition, our data implies that inhibition of miR-21 is a novel therapeutic strategy in flap surgeries although the detail mechanism is currently unclear. The proposed model is shown in Figure 6.
